# Automated abdominal adipose tissue segmentation and volume quantification on longitudinal MRI using 3D convolutional neural networks with multi-contrast inputs

**DOI:** 10.1007/s10334-023-01146-3

**Published:** 2024-02-01

**Authors:** Sevgi Gokce Kafali, Shu-Fu Shih, Xinzhou Li, Grace Hyun J. Kim, Tristan Kelly, Shilpy Chowdhury, Spencer Loong, Jeremy Moretz, Samuel R. Barnes, Zhaoping Li, Holden H. Wu

**Affiliations:** 1 Department of Radiological Sciences, University of California, 300 UCLA Medical Plaza, Suite B119, Los Angeles, CA 90095 USA; 2 Department of Bioengineering, University of California, Los Angeles, CA USA; 3 Department of Physiological Science, University of California, Los Angeles, CA USA; 4 Department of Radiology, Loma Linda University Medical Center, Loma Linda, CA USA; 5 Department of Psychology, Loma Linda University School of Behavioral Health, Loma Linda, CA USA; 6 Department of Neuroradiology, Loma Linda University Medical Center, Loma Linda, CA USA; 7 Department of Medicine, University of California, Los Angeles, CA USA

**Keywords:** Adipose tissue, Body composition, Neural networks, Overweight, Obesity, Automated segmentation, Magnetic resonance imaging

## Abstract

**Objective:**

Increased subcutaneous and visceral adipose tissue (SAT/VAT) volume is associated with risk for cardiometabolic diseases. This work aimed to develop and evaluate automated abdominal SAT/VAT segmentation on longitudinal MRI in adults with overweight/obesity using attention-based competitive dense (ACD) 3D U-Net and 3D nnU-Net with full field-of-view volumetric multi-contrast inputs.

**Materials and methods:**

920 adults with overweight/obesity were scanned twice at multiple 3 T MRI scanners and institutions. The first scan was divided into training/validation/testing sets (n = 646/92/182). The second scan from the subjects in the testing set was used to evaluate the generalizability for longitudinal analysis. Segmentation performance was assessed by measuring Dice scores (DICE-SAT, DICE-VAT), false negatives (FN), and false positives (FP). Volume agreement was assessed using the intraclass correlation coefficient (ICC).

**Results:**

ACD 3D U-Net achieved rapid (< 4.8 s/subject) segmentation with high DICE-SAT (median ≥ 0.994) and DICE-VAT (median ≥ 0.976), small FN (median ≤ 0.7%), and FP (median ≤ 1.1%). 3D nnU-Net yielded rapid (< 2.5 s/subject) segmentation with similar DICE-SAT (median ≥ 0.992), DICE-VAT (median ≥ 0.979), FN (median ≤ 1.1%) and FP (median ≤ 1.2%). Both models yielded excellent agreement in SAT/VAT volume versus reference measurements (ICC > 0.997) in longitudinal analysis.

**Discussion:**

ACD 3D U-Net and 3D nnU-Net can be automated tools to quantify abdominal SAT/VAT volume rapidly, accurately, and longitudinally in adults with overweight/obesity.

**Supplementary Information:**

The online version contains supplementary material available at 10.1007/s10334-023-01146-3.

## Introduction

Adipose tissue (AT) is a long-term energy depot that can release fatty acids to satisfy the body’s energy needs [1]. Increased AT volume is highly associated with escalating incidences of overweight/obesity [2]. There are two main compartments of AT: visceral adipose tissue (VAT) and subcutaneous adipose tissue (SAT). In previous longitudinal studies, abdominal SAT and VAT have been found to be associated with risks of various cardiometabolic diseases such as high blood pressure and high glucose [3–7]. In particular, VAT is related to hormonal activity through the release of adipokines [8] and therefore plays a key role in metabolic activity via secretion of inflammatory markers [9]. Adipokines and proinflammatory cytokines secreted by VAT are contributing causes to the development of obesity-related tumors [10]. To reduce the burden of these metabolic disorders, dietary interventions can be beneficial [11]. Longitudinal quantification of SAT and VAT volume can be crucial for monitoring [6, 12].

Magnetic resonance imaging (MRI) is a powerful imaging modality that can accurately quantify SAT and VAT [13–15]. Currently, the reference standard to quantify SAT/VAT is based on manual annotations [9, 13], which require expert knowledge and are time-consuming and challenging for large-scale and longitudinal studies [16, 17]. These challenges are especially prominent while annotating VAT due to its spatially complex nature. A rapid and accurate technique to segment SAT and VAT using MRI is needed to facilitate research and clinical applications, such as longitudinal monitoring of SAT/VAT volume in subjects with overweight/obesity.

Because of the clinical importance of SAT and particularly VAT, several studies proposed machine/deep learning approaches to segment SAT and VAT using MRI [16, 18–24]. Recently, deep learning-based methods that are based on convolutional neural networks (CNNs) have been proposed, using 2-dimensional (2D) neural network architectures such as 2D U-Net [22, 23] and 2D Dense U-Net [25]. FatSegNet, a 2.5D approach, implemented an aggregation neural network to combine 2D segmentations obtained from images in 3 different orientations (i.e., axial, sagittal, coronal) and achieved Dice scores of around 0.85 for VAT in the abdomen [16]. Another study compared 2D U-Net with 3-dimensional (3D) U-Net and concluded that given the same training data, 3D U-Net based neural networks outperformed the 2D U-Net especially for VAT segmentation in the abdomen [17]. Küstner et al. proposed 3D Densely Connected Net [24] to automatically segment SAT and VAT, and achieved whole-body VAT segmentation with mean Dice scores of 0.89 in 300 adults. The previous literature using CNNs reported excellent SAT segmentation performance (Dice scores of 0.97–0.99), but VAT segmentation performance levels varied markedly (Dice scores of 0.43–0.89) due to its complex spatially varying nature [16, 22–24]. For instance, 2D CNNs can suffer from the inability to consider the 3D anatomical through-plane associations [22, 23, 25]. The 3D Densely Connected Net utilized 3D image patch inputs yet image patches still might not fully capture the global in-plane and through-plane associations across the full field-of-view (FOV) [24]. Lastly and most importantly, previous studies did not test the generalization capability of their proposed CNNs for longitudinal MRI data.

To address the problems associated with inability to fully capture the global in-plane and through-plane associations in VAT, recent work [17, 26] using 3D U-Net and attention-based competitive dense (ACD) 3D U-Net with full-FOV 3D volumetric multi-contrast Dixon MRI inputs and a novel frequency balancing boundary emphasizing Dice loss (FBDL) showed promising results (Dice score for abdominal SAT > 0.97 and Dice score for abdominal VAT > 0.94). Further investigation is required to characterize the contributions of specific components in ACD 3D U-Net and test its performance in a larger dataset.

In addition, nnU-Net, a CNN based on a U-Net-like network architecture, has been recently proposed as an out-of-the-box tool that can excel at various segmentation tasks in medical imaging [27]. Compared to U-Net, nnU-Net has advantages of self-configuration of parameters such as network architecture, patch size, and batch size after considering input dataset properties and pipeline fingerprints. nnU-Net has found success in a range of applications such as automated breast segmentation [28], brain tumor segmentation [29], pancreatic fat segmentation [30], fetal SAT segmentation [31], and SAT/VAT segmentation [32] on MRI. The previous work on automated SAT/VAT segmentation in the body trunk achieved Dice scores > 0.94 for both SAT and VAT in a cross-validation study of 30 adult subjects [32]. Further investigation is needed to assess the generalizability of nnU-Net in a larger testing dataset and in a longitudinal study.

The objectives of this study were to (a) further develop ACD 3D U-Net and adapt 3D nnU-Net for rapid abdominal SAT and VAT segmentation and quantification using a larger Dixon MRI dataset in adults with overweight/obesity, and (b) assess the generalization capability of ACD 3D U-Net and 3D nnU-Net to analyze longitudinal MRI by testing in data from a subsequent MRI scan without further training.

## Materials and methods

### Study design

This HIPAA-compliant and IRB-approved study performed a new retrospective analysis of data from the Habitual Diet and Avocado Trial (“HAT”), which was conducted from June 2018 to March 2020 under the clinical trial number NCT03528031 [11, 33]. HAT is a multi-center, randomized, controlled study designed to test whether providing one avocado per day for consumption for six months compared to a habitual diet essentially devoid of avocados will result in a decrease in visceral adiposity in adults with overweight [11, 33]. Inclusion criteria were: age ≥ 25 years old, waist circumference ≥ 35 inches (86 cm) for women and ≥ 40 inches (101 cm) for men, and regular consumption of ≤ 2 avocados per month. HAT enrolled 1008 adults [33]. All subjects provided written informed consent, and 923 subjects successfully completed MRI at two time points. The same HAT data were analyzed in different contexts in previous publications [11, 17, 26, 33]. This work focused on retrospective image analysis to evaluate 3D convolutional neural networks for abdominal SAT and VAT segmentation and quantification on MRI at two time points.

### Data preparation

The 923 subjects underwent abdominal MRI scans on seven different 3 T scanners (Skyra or Prisma, Siemens Healthineers, Erlangen, Germany) at five imaging centers at baseline (first MRI scan) and at 6 months (second MRI scan). Each subject was brought back to the same MRI scanner for the second scan. A consistent acquisition protocol was used at all imaging centers. An axial T_1_-weighted 3D dual-echo (in-phase echo-time image, TE^IP^; opposed-phase echo-time image, TE^OP^) Dixon MRI sequence was acquired to calculate water (W) and fat (F) images [11, 33, 34] **(**Table 1**)**. We excluded one subject from the HAT data due to local fat/water swaps in the first MRI scan. For the second MRI scan, two additional subjects were excluded due to data formatting issues. Therefore, n = 920 subjects were included for analysis in this work. All data were deidentified using research record numbers.

**Table 1:** Magnetic resonance imaging (MRI) parameters

T_1_-Weighted 3D Dual-Echo Dixon MRI (Axial)	
Magnetic field strength	3 T
Echo time (TE)	1.23 ms (TE^OP^), 2.46 ms (TE^IP^)
Repetition time (TR)	5 ms
Flip angle	9 ^∘^
Matrix size	192 $\times$ 192
Field-of-View (FOV)	400 mm $\times$ 400 mm
In-plane resolution	2.08 mm $\times$ 2.08 mm
Slice thickness	5 mm
Number of acquired slices	96
Number of analyzed slices	51
Scan time	17 s

In each subject in the Habitual Diet and Avocado Trial (HAT), 96 MRI slices were acquired to cover 4 cm above the dome of the liver to 7 cm below the top of the iliac crest. For analysis of abdominal adipose tissue in the HAT, 51 slices were chosen to start from the iliac crest as a reference landmark and include 50 slices in the superior direction. See the text for details

*OP* Opposed-phase, *IP* In-phase

### Reference segmentation

This study is a retrospective analysis based on the HAT MRI data. In each subject in the HAT, 96 slices were acquired to cover 4 cm above the dome of the liver to 7 cm below the top of the iliac crest. For the study purposes of HAT, 51 contiguous axial slices (spanning 255 mm in the superior-inferior [S/I] direction) were chosen in the abdomen for reference segmentation of abdominal SAT and VAT. A trained researcher (five years of experience) selected these 51 slices by identifying the iliac crest as a landmark for the inferior limit and counting 50 slices in the superior direction. The choice of 51 slices was designed to include as many slices that are free of artifacts as possible, as with large slice coverage the image quality degrades in slices towards the top and bottom of the volume, with swapping of the fat/water signal, fall off in the signal-to-noise ratio, and other problems. The goal for MRI analysis in HAT was to include as many slices as possible in a consistent manner without having to exclude datasets because of poor image quality.

Reference segmentation of abdominal SAT and VAT was performed by the trained researcher on the Dixon fat images in a semi-automated manual approach using SliceOmatic (TomoVision, Montreal, Canada) with a watershed algorithm and ImageJ (National Institute of Health) was used for thresholding using Otsu algorithm. Thresholded results were reviewed by the researcher and any non-VAT pixels (such as from the vertebral bodies or bone marrow) were manually removed [11, 33]. An attending radiologist specializing in body imaging (ten years of experience) supervised the trained researcher during the reference segmentation process. Note that the slice selection and reference segmentation of abdominal SAT/VAT were originally completed for the purposes of the HAT and subsequently used in this study to train and evaluate 3D CNNs for segmentation.

### Data partitioning

We stratified the data from the first MRI scan (n = 920 subjects) into separate training (70%, n = 646), validation (10%, n = 92), and testing (20%, n = 182) sets with similar body-mass index (BMI) and age (Table 2) to investigate 3D CNNs for segmenting abdominal SAT/VAT. The female to male ratios across training, validation, and testing datasets were also similar. Other demographic information such as race/ethnicity were reported in [11]. Our study included two testing sets that are separate from training and validation data. The first testing set included 182 subjects from the same time point as the training and validation sets (first MRI scan), and the second testing set consisted of the corresponding second MRI scans (6-months after the first scan) from the 182 subjects in the first testing set. The main purpose of the second testing set was to evaluate the generalizability of the 3D CNNs (trained at the initial time point) to subsequent time points without further training. All testing was performed with respect to the reference segmentation at the corresponding time point. Both testing sets allowed us to evaluate the segmentation performance across different BMI.

**Table 2:** Demographic information for subjects with overweight or obesity (n = 920)

	All	Training	Validation	Testing
Number of subjects	n = 920	n = 646	n = 92	n = 182
Age*	50.4 $\pm$ 14.0 years	51.1 $\pm$ 14.0 years	47.6 $\pm$ 13.1 years	49.7 $\pm$ 14.2 years
Sex^a^	677 F, 243 M	487 F, 159 M	70 F, 22 M	120 F, 62 M
BMI*	32.9 $\pm$ 5.5 kg/m^2^	32.8 $\pm$ 5.5 kg/m^2^	32.8 $\pm$ 5.5 kg/m^2^	33.2 $\pm$ 5.5 kg/m^2^

The age and body mass index (BMI) of the subjects are reported as a mean ± standard deviation. The training, validation, and testing sets refer to data from the first MRI scan (baseline)

*F* Female, *M* Male

^*^No statistically significant differences were found among training, validation, and testing datasets according to Kruskal–Wallis’s test (p > 0.05)

^a^The female to male ratios across training, validation, and testing datasets were similar

### 3D convolutional neural network models

**Fig. 1:**
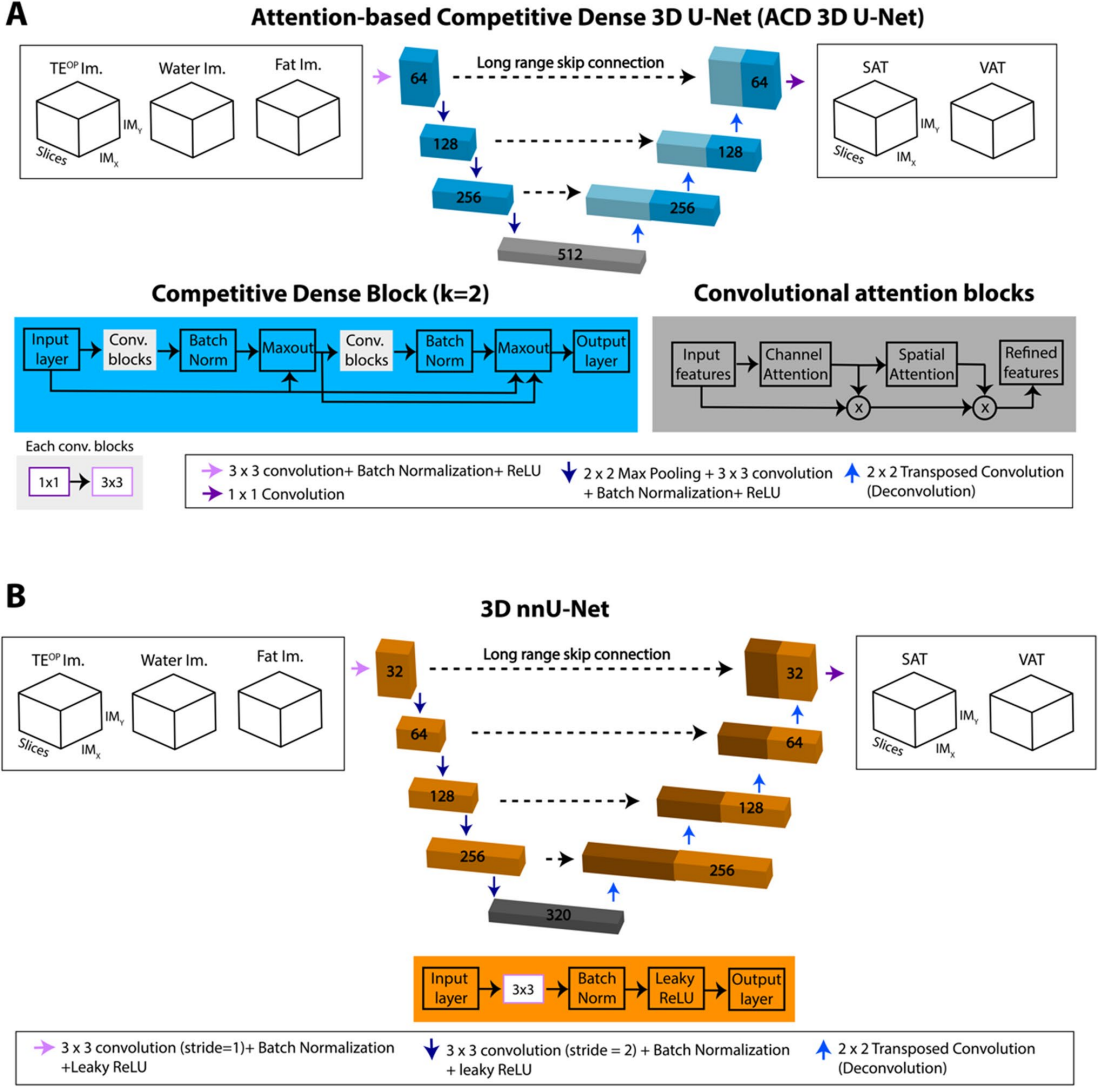
The network structures for **A** attention-based competitive dense (ACD) 3D U-Net and **B** 3D nnU-Net. The filter sizes are reported on top of each convolutional block. The full field-of-view volumetric opposed-phase echo image (TE^OP^), water, and fat images were combined as the multi-contrast inputs, and the 3D networks outputted segmentation masks for abdominal SAT and VAT concurrently. **A** In the ACD 3D U-Net, each blue box represents a competitive densely connected block. At the bottom layer, convolutional attention blocks shown in dark gray color were added to consider the more informative spatial and channel features. **B** A self-configuring 3D nnU-Net was trained based on a previously published framework. At each resolution step, 3D nnU-Net employed a convolution, followed by instance normalization and a leaky rectified linear unit (ReLU) activation function. Strided convolution and transposed convolution were used for downsampling and upsampling, respectively

First, we trained 3D U-Net [35] with weighted Dice loss (WDL) [23, 36] and full-FOV volumetric opposed-phase (TE^OP^), W, and F images to exploit the multi-contrast MRI information [17]. In a full FOV scheme, 3D volumetric images are inputted to 3D CNNs as a whole, as opposed to 2D or 3D patch-based inputs extracted from the original images. In other words, full-FOV can be defined as all the slices between two landmarks (e.g., all axial images between a set of S/I landmarks). Next, we trained ACD 3D U-Net [17], which leverages competitive dense connections [37, 38] and channel-spatial attention blocks [39] (Fig. 1A). Competitive dense connection**s** are effective for learning features with a small training sample size with high computational efficiency. The filter sizes were 64, 128, 256, and 512 at the bottom layer [35, 40]. The bottom layer included a convolutional attention block to focus on more informative spatial and channel features. While training the ACD 3D U-Net, FBDL was used to address the complex features of VAT and the class imbalance between SAT and VAT [17, 26, 41]. For 3D U-Net and ACD 3D U-Net, all image volumes were normalized to have pixel values between 0 and 255. Gradients were zeroed out before each batch. Weights for the CNNs were initialized randomly. Hyperparameters such as number of epochs and learning rate were optimized while minimizing the loss function in the validation dataset using the Adam optimizer without data augmentation.

In addition, we trained 3D nnU-Net, a self-configuring neural network based on 3D U-Net [27] (Fig. 1B). 3D nnU-Net was automatically configured according to a previous framework using the full-resolution version of 3D nnU-Net [27]. For full-resolution 3D nnU-Net, the patch size is automatically set as the biggest patch size that fits into the graphical processing unit (GPU) with a minimum batch size of 2. In our study, the patch size was determined to be the full input image size. Therefore, 3D nnU-Net used in our study is a full-FOV CNN that processes the entire input image volume at once. In 3D nnU-Net, ReLU nonlinearities were replaced with leaky rectified linear unit (ReLUs) (negative slope, 0.01) in contrast to 3D U-Net. Strided convolution and transposed convolution were used for downsampling and upsampling processes, respectively. Per the originally published 3D nnU-Net framework, the initial filter size was set to 32 and doubled (halved) with each downsampling (upsampling) operation. The maximum filter size was capped at 320 instead of 512 to limit the memory consumption. Nesterov momentum (µ = 0.99) and an initial learning rate of 0.01were used for training 3D nnU-Net. Each input image was normalized by subtracting the mean, and then dividing by its standard deviation.

All 3D CNNs were implemented in PyTorch 1.8.0 and trained on a NVIDIA Quadro RTX 8000 GPU with 48 GB memory. The code for implementing the 3D U-Net and ACD 3D U-Net and the final weights for all the trained 3D CNNs are available at https://github.com/HoldenWuLab/AT_Seg_3DCNN.

### Ablation study: ACD 3D U-Net

We performed an ablation study to investigate three main aspects of ACD 3D U-Net using the first testing set (n = 182): *Network structures* (3D U-Net vs. ACD 3D U-Net), *Loss functions* (WDL vs. FBDL), and *Network inputs* (TE^OP^ + TE^IN^ vs. W + F vs. TE^OP^ + W + F), resulting in five CNNs (Supplementary Table 1). After the ablation study, we choose two representative CNNs for further analysis: (a) 3D U-Net with WDL and TE^OP^ + W + F and (b) ACD 3D U-Net with FBDL and TE^OP^ + W + F. For the sake of clarity, we will refer to the latter as the proposed ACD 3D U-Net.

### Ablation study: 3D nnU-Net

By using the input images (TE^OP^ + W + F) that were shown to be beneficial for ACD 3D U-Net, we investigated the effects of loss functions (WDL vs. FBDL) on 3D nnU-Net performance in a separate ablation study (Supplementary Table 1) using the testing set of the first MRI (n = 182).

### Evaluation of segmentation performance

We evaluated the abdominal SAT/VAT segmentation performance with respect to reference segmentations (semi-automated manual annotations) in the first testing set (n = 182, first MRI) and the second testing set (n = 182, second MRI) in terms of 3D Dice scores for VAT and SAT (DICE-VAT, DICE-SAT), false negatives (FN) [%], and false positives (FP) [%] per subject [42], which were then reported as median and interquartile range (IQR) across subjects in the testing sets. FP and FN considered SAT and VAT together (Eqs. 1 and 2).

1 $$FP=100\%\times \frac {{\sum }_{l=1}^{SAT, VAT}{\sum }_{n=1}^{N}I({r}_{ln}==0)\cap I( {p}_{ln}==1)}{{\sum }_{l=1}^{SAT, VAT}{\sum }_{n=1}^{N}I({r}_{ln}==0)}$$

2 $$FN=100\%\times \frac {{\sum }_{l=1}^{SAT, VAT}{\sum }_{n=1}^{N}I({r}_{ln}==1)\cap I( {p}_{ln}==0)}{{\sum }_{l=1}^{SAT, VAT}{\sum }_{n=1}^{N}I({r}_{ln}==1)}$$

$l$ represents the classes, ${r}_{ln}$ and ${p}_{ln}$ denote the reference (0 when the pixel does not include the corresponding target reference segmentation mask, e.g., SAT or VAT) and output segmented pixel $n$, and $I$ is the indicator function.

### Evaluation of volume quantification

We evaluated 3D U-Net, the proposed ACD 3D U-Net, and 3D nnU-Net for abdominal SAT/VAT volume quantification with respect to reference annotations in the first and second testing sets using linear regression and the Intraclass Correlation Coefficient (ICC) [43], as ICC has been used in previous work [16, 19]. ICC values below 0.5 indicate poor agreement, between 0.5 and 0.75 indicate moderate agreement, between 0.75 and 0.9 indicate good agreement, and any value above 0.9 indicates excellent agreement [44]. The changes in the SAT and VAT volume between two MRI scans ($\Delta$ Volume _AT_ = Volume2_AT_ – Volume1_AT_ where AT can be SAT or VAT) measured by reference annotations and the 3D CNNs were also evaluated by linear regression.

### Statistical analysis

For the ablation study of the ACD 3D U-Net using the first MRI scans, group-wise segmentation performance differences in terms of DICE-SAT, DICE-VAT, FN, and FP across the five CNNs were first compared by using a Kruskal–Wallis test. Then, Wilcoxon Signed-Rank tests were performed to evaluate the pairwise differences between the proposed ACD 3D U-Net and the four other CNNs in the ablation study (four pairs). Based on the ablation study results, we selected the proposed ACD 3D U-Net and 3D U-Net for further analysis in the second MRI.

For the ablation study of the 3D nnU-Net using the first MRI scans, a Wilcoxon Signed-Rank test was used to evaluate the pair-wise differences between 3D nnU-Net trained with WDL or FBDL. Based on the resultant performance levels, we selected 3D nnU-Net with WDL for further analysis.

To analyze the results in the testing set of the second MRI, we first performed a Kruskal–Wallis test across the results from ACD 3D U-Net, 3D U-Net, and 3D nnU-Net. Then, the differences in segmentation performance (DICE-SAT, DICE-VAT, FN, and FP) were compared using Wilcoxon Signed-Rank tests for pairwise comparisons.

For all tests (first and second MRI), the Benjamini–Hochberg procedure was used for multiple comparisons to control the false discovery rate (FDR) to a level of 0.05 [45].

## Results

### Subject demographics

Subject demographics from the 920 subjects included in this study are reported in Table 2. Our study cohort consisted of 677 females and 243 males. The age (mean $\pm$ standard deviation [SD]) was 50.4 $\pm$ 14.0 years and the BMI (mean $\pm$ SD) was 32.9 $\pm$ 5.5 kg/m^2^. The range of age was [25.1, 86.6] years and the range of BMI was [20.7, 60.4] kg/m^2^. For the first MRI scan, the mean age and BMI were kept similar across training (70%, n = 646), validation (10%, n = 92) and testing (20%, n = 182) sets. The female to male ratios across datasets were also similar.

### 3D convolutional neural network training and inference

Training/inference time and the number of trainable parameters for 3D U-Net, the proposed ACD 3D U-Net, and 3D nnU-Net are reported in Table 3. Supplementary Table 1 summarizes the extended analysis of 3D CNNs in the two ablation studies. The proposed ACD 3D U-Net was trained in ~ 32.9 h, while 3D U-Net had a shorter training time of 22.6 h. 3D nnU-Net, on the other hand, took the longest to train (66.6 h using WDL). ACD 3D U-Net had fewer trainable parameters than 3D U-Net and 3D nnU-Net. The inference times were rapid for all 3D CNNs: < 75 ms/slice (i.e., < 4.8 s per 3D volume) for 3D U-Net and ACD 3D U-Net, and 36 ms/slice (< 2.5 s per 3D volume) for 3D nnU-Net.

**Table 3:** Training time, inference time, and number of trainable parameters for the tested 3D convolutional neural networks

Network	Loss function	Inputs	Training time (h)	Inference time (ms/slice)	Trainable parameters (M)
3D U-Net	WDL	TE^OP^ + W + F	22.6	65	~ 22.4
ACD 3D U-Net	FBDL	TE^OP^ + W + F	32.9	72	~ 20.8
3D nnU-Net	WDL	TE^OP^ + W + F	66.6	36	~ 30.0

*ACD* Attention-based competitive dense, *WDL* weighted Dice loss, *FBDL* frequency-balancing boundary-emphasizing Dice loss, *TE*^*OP*^ opposed-phase image, *W* water image, *F* fat image

### Representative examples

**Fig. 2:**
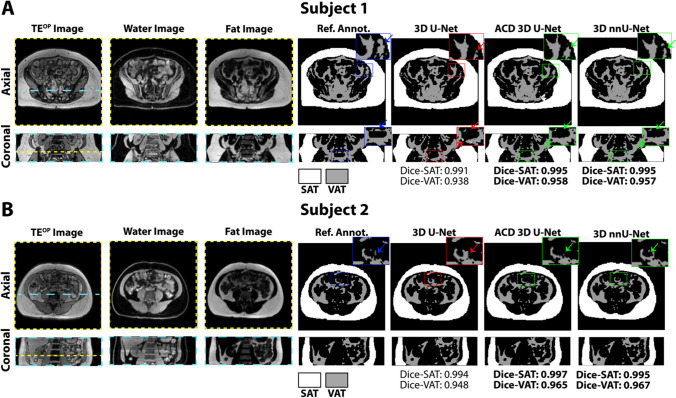
Input images (TE^OP^, fat, and water), reference annotations, and output abdominal SAT (white) and VAT (gray) segmentation masks from 3D U-Net, attention-based competitive dense (ACD) 3D U-Net, and 3D nnU-Net in two representative subjects. **A** Results for subject 1, a 75-year-old female with body-mass-index (BMI) of 29.0 kg/m^2^. **B** Results for subject 2, a 41-year-old female with BMI of 30.2 kg/m^2^. The regions misclassified by 3D U-Net were shown in zoomed insets and indicated by red arrows. The proposed ACD 3D U-Net and 3D nnU-Net were able to segment the corresponding regions (green arrows) with closer agreement to the reference annotations (Ref. Annot.). The highest DICE scores of the three CNNs were indicated with bold font

**Fig. 3:**
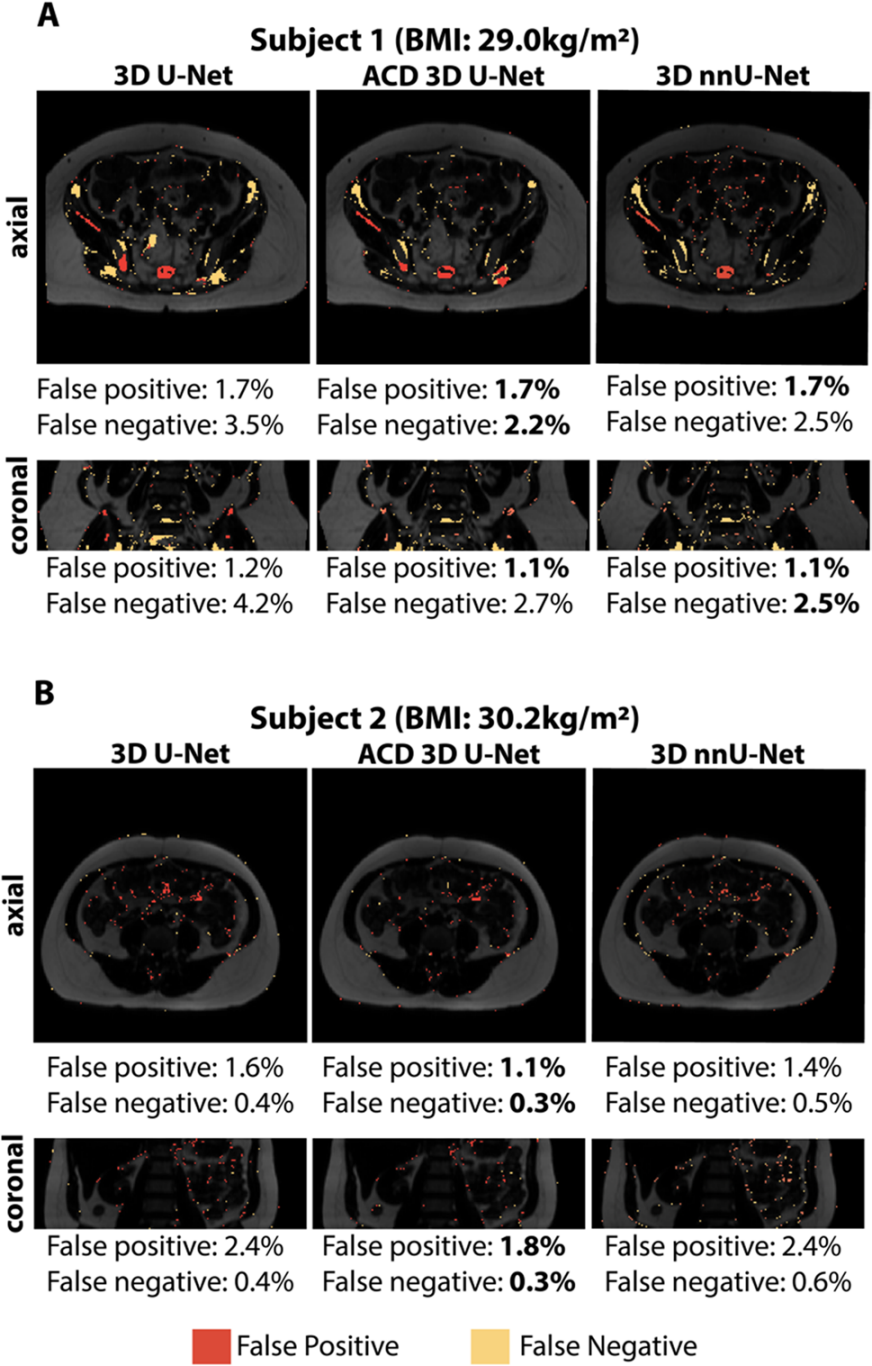
False negative pixels (yellow) and false positive pixels (red) for the two representative subjects in Fig. 2 (**A**, **B**) using 3D U-Net, the proposed attention-based competitive dense (ACD) 3D U-Net, and 3D nnU-Net. The false negative and positive ratios are reported for the shown slices and the lowest values are highlighted with bold font

Representative images from a 75-year-old female with BMI of 29.0 kg/m^2^ and a 41-year-old female with BMI of 30.2 kg/m^2^ are shown in Fig. 2. The multi-contrast MRI inputs (TE^OP^, W, and F images) together with the reference annotations and 3D CNN outputs (SAT and VAT masks) are depicted in axial and reformatted coronal views. The proposed ACD 3D U-Net yielded higher 3D DICE-SAT and 3D DICE-VAT scores than those of 3D U-Net for both subjects. 3D nnU-Net yielded similar 3D DICE-SAT and DICE-VAT scores as ACD 3D U-Net. The FN pixels (yellow) and FP pixels (red) for the same representative subjects are shown in Fig. 3. The proposed ACD 3D U-Net generally achieved fewer FN and FP pixels than 3D U-Net and 3D nnU-Net for both representative subjects. 3D-rendered depictions of abdominal SAT/VAT segmentations along with FN (yellow) and FP (red) pixels using the proposed ACD 3D U-Net and 3D nnU-Net are shown in Supplementary Videos 1 and 2, respectively.

### Evaluation of segmentation performance using the first MRI scan

The distributions of DICE-SAT, DICE-VAT, FN, and FP in the first testing set (n = 182) are shown in Supplementary Figs. 1 and 2 for all five CNNs in the ablation study for the ACD 3D U-Net. The group-wise differences between all five CNNs were significant for DICE-SAT (p < 0.001), DICE-VAT (p < 0.001), FN (p < 0.001), and FP (p < 0.001). The highest median Dice scores (DICE-SAT: 0.994, DICE-VAT: 0.976), as well as the highest minimum Dice scores (DICE-SAT: 0.935, DICE-VAT: 0.825) scores, were achieved by the proposed ACD 3D U-Net with FBDL and TE^OP^ + W + F. The highest FN and FP values were produced by the ACD 3D U-Net with FBDL and TE^OP^ + TE^IN^. The overall best performing CNN was the proposed ACD 3D U-Net with FBDL and TE^OP^ + W + F, which achieved significantly higher DICE-SAT and DICE-VAT scores (p < 0.001) and significantly smaller FN values (p < 0.001) than the other CNNs.

In the ablation study for 3D nnU-Net, the corresponding DICE-SAT, DICE-VAT, FN and FP distributions are shown in Supplementary Fig. 3. Overall segmentation performance was similar for 3D nnU-Net with WDL and FBDL. 3D DICE-VAT was significantly higher for WDL than FBDL (p < 0.001), and FN for WDL was significantly smaller than those of FBDL (p < 0.001). Additionally, considering the shorter training time, we chose 3D nnU-Net with WDL for further analysis.

**Fig. 4:**
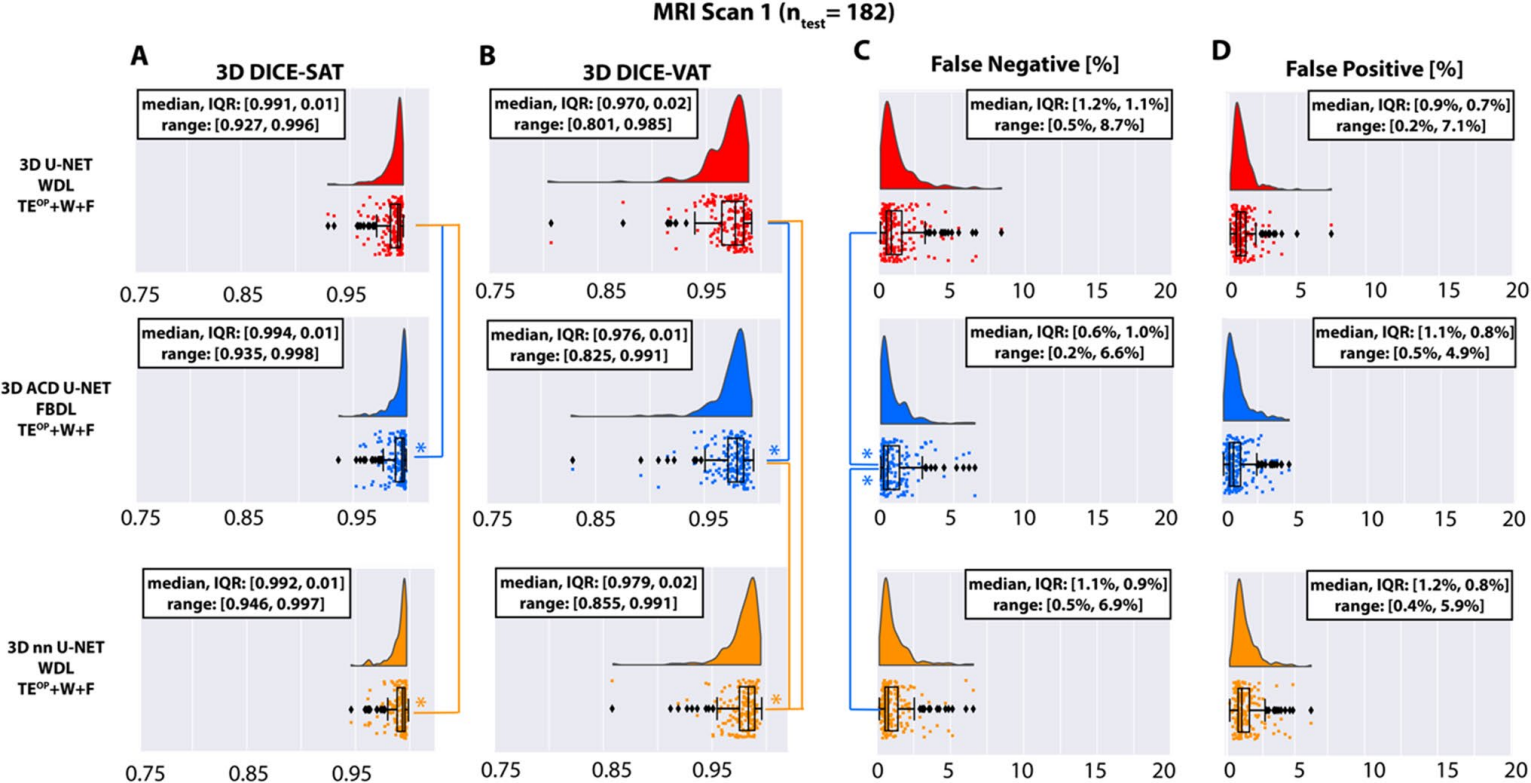
Segmentation performance of the 3D U-Net, the proposed attention-based competitive dense (ACD) 3D U-Net, and 3D nnU-Net in terms of the 3D DICE-SAT (**A**), DICE-VAT (**B**), false negatives (FN) (**C**), and false positives (FP) (**D**) in the testing set of the first MRI (n = 182). The * indicates statistically significant differences in median scores using Benjamini-Hochberg’s procedure for multiple comparison. The color of * represents the better performing 3D CNN. Overall, ACD 3D U-Net and 3D nnU-Net performed similarly and were better than 3D U-Net. Median DICE-SAT (p < 0.001) and DICE-VAT (p < 0.001) were significantly higher for 3D nnU-Net and ACD 3D U-Net than 3D U-Net. ACD 3D U-Net achieved significantly lower median FN when compared to 3D U-Net and 3D nnU-Net (p < 0.001). ACD 3D U-Net produced lower maximum FP values than 3D U-Net and 3D nnU-Net

Figure 4 shows the segmentation performance of 3D U-Net, the proposed ACD 3D U-Net, and 3D nnU-Net in the first testing set. Compared to 3D U-Net, significantly higher median DICE-SAT (p < 0.001) and median DICE-VAT (p < 0.001) were achieved by the proposed ACD 3D U-Net (0.994 and 0.976) and 3D nnU-Net (0.992 and 0.979). The proposed ACD 3D U-Net produced higher minimum DICE-SAT (0.935 versus 0.927) and DICE-VAT (0.825 versus 0.801) than 3D U-Net. 3D nnU-Net produced the highest minimum DICE-SAT (0.946) and DICE-VAT (0.855). The proposed ACD 3D U-Net yielded significantly smaller median FN than 3D U-Net (0.6% versus 1.2%, p < 0.001) and 3D nnU-Net (0.6% versus 1.1%, p < 0.001). The maximum FN was also reduced for the proposed ACD 3D U-Net (6.6%) when compared to 3D nnU-Net (6.9%) and 3D U-Net (8.7%). The median FP for the three networks were not significantly different. The maximum FP produced by the proposed ACD 3D U-Net (4.9%) was smaller than 3D nnU-Net (5.9%) and 3D U-Net (7.1%).

### Evaluation of segmentation performance using the second MRI scan

**Fig. 5:**
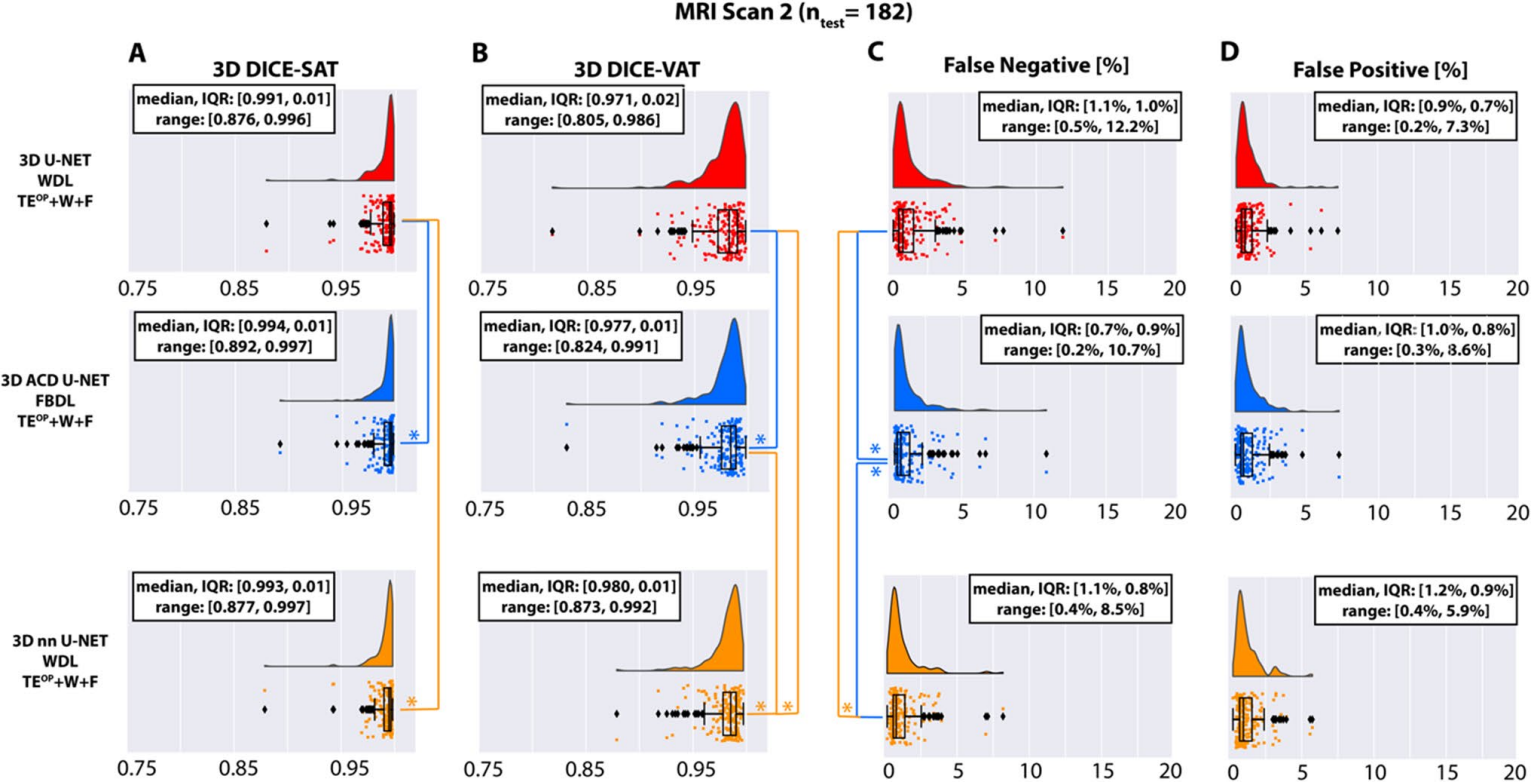
Segmentation performance of the 3D U-Net, the proposed attention-based competitive dense (ACD) 3D U-Net, and 3D nnU-Net in terms of the 3D DICE-SAT (**A**), DICE-VAT (**B**), false negatives (FN) (**C**), and false positives (FP) (**D**) in the testing set of the second MRI (n = 182). The * indicates statistically significant differences in median scores using Benjamini-Hochberg’s procedure for multiple comparison. The color of * represents the better performing 3D CNN. Overall, ACD 3D U-Net and 3D nnU-Net performed similarly and were better than 3D U-Net. ACD 3D U-Net and 3D nnU-Net achieved significantly higher median DICE-SAT (p < 0.001) and DICE-VAT (p < 0.001) scores compared to 3D U-Net. ACD 3D U-Net achieved significantly lower median FN when compared to 3D U-Net and 3D nnU-Net (p < 0.001). 3D nnU-Net produced lower maximum FP values than 3D U-Net and ACD 3D U-Net

The distributions of DICE-SAT, DICE-VAT, FN, and FP in the second MRI scan (n = 182) without further training are shown in Fig. 5. The proposed ACD 3D U-Net achieved higher minimum DICE-SAT (0.892 versus 0.876 for 3D U-Net and 0.877 for 3D nnU-Net). 3D nnU-Net yielded the highest minimum DICE-VAT (0.873 versus 0.824 for ACD 3D U-Net and 0.805 for 3D U-Net) **(**Fig. 5B**)**. The median DICE-SAT (0.994 versus 0.991, p < 0.001) and DICE-VAT (0.977 versus 0.971, p < 0.001) were significantly higher for the proposed ACD 3D U-Net compared to 3D U-Net. The median DICE-VAT for 3D nnU-Net (0.980) was significantly higher than the other 2 CNNs (p < 0.001). The median FN were significantly lower (p < 0.001) for the proposed 3D ACD U-Net (0.7%) than that for 3D U-Net (1.1%) and 3D nnU-Net (1.1%) (Fig. 5C). The maximum FN was also reduced for 3D nnU-Net (8.5%) when compared to the proposed ACD 3D U-Net (10.7%) and 3D U-Net (12.2%). The median FP for the three networks were not significantly different.

### Evaluation of volume quantification using the first and second MRI scans

**Fig. 6:**
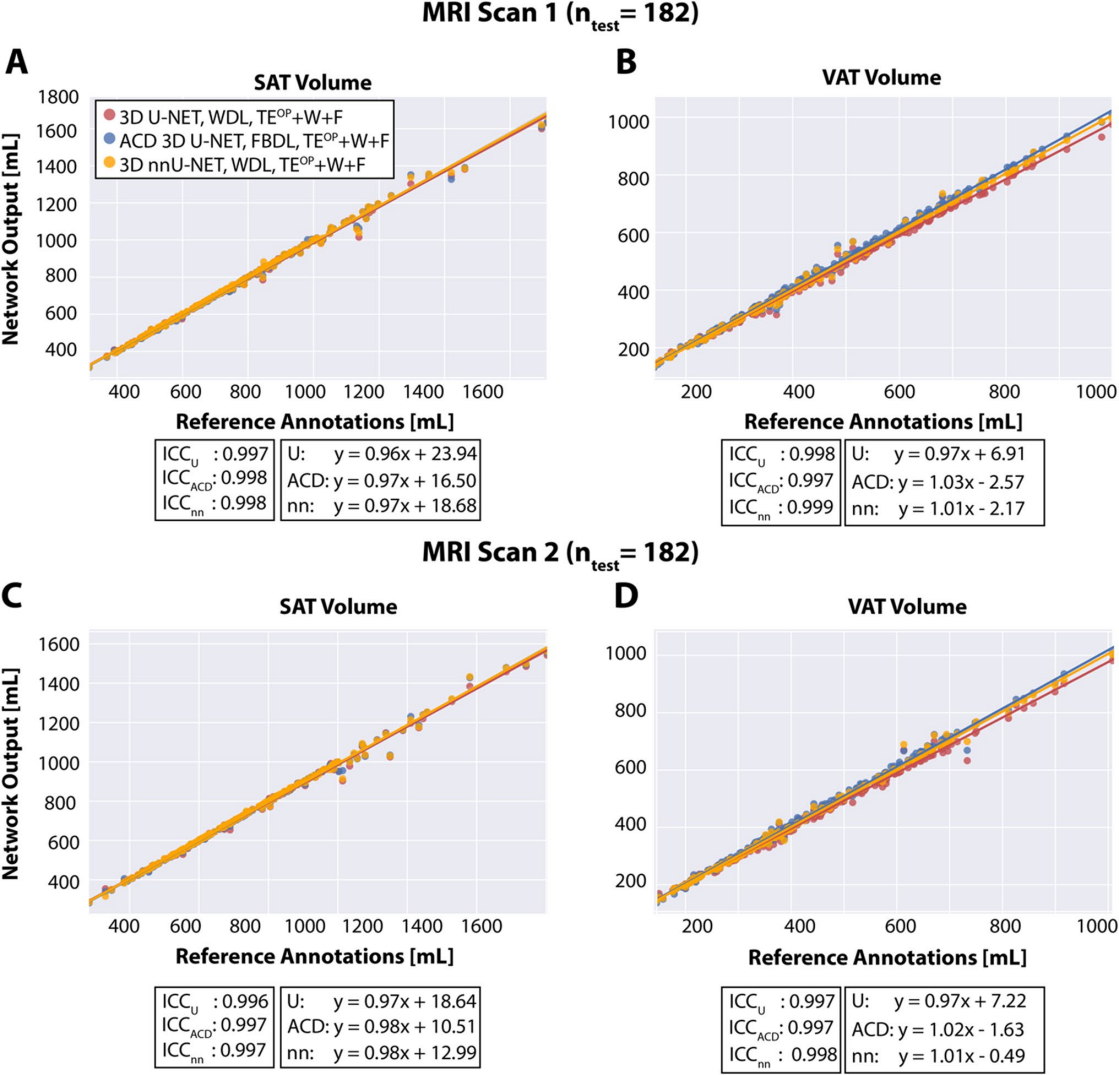
The abdominal subcutaneous and visceral adipose tissue (SAT, VAT) volume agreement between the reference annotations and results from 3D U-Net, ACD 3D U-Net, and 3D nnU-Net. **A**, **B** Results from the testing set of the first MRI scan (n = 182). **C**, **D** Results from the testing set of the second MRI scan (n = 182). The 3D convolutional neural networks trained with the same 646 subjects from the first MRI. The linear regression equations as well as intra-class correlation coefficients (ICC) are reported in the figure legends. *ICC*_*U*_ ICC value for the 3D U-Net. *ICC*_*ACD*_ ICC value for ACD 3D U-Net. *ICC*_*nn*_ ICC value for 3D nnU-Net

Figure 6 shows the abdominal SAT/VAT volume quantification results for 3D U-Net, the proposed ACD 3D U-Net, and 3D nnU-Net using the first and second MRI scans from subjects in the testing set. The slopes of the linear regression (range: [0.96, 1.03]) and the ICC $\ge$ 0.997 for the testing set of the first MRI (n = 182) demonstrate that 3D U-Net, the proposed ACD 3D U-Net, and 3D nnU-Net achieved excellent agreement in abdominal SAT/VAT volume with respect to the reference annotations. When the second MRI scans were used as a separate testing set (n = 182) without further training, the volume agreement was also excellent with ICC $\ge$ 0.997 and slopes near unity in the range of [0.98, 1.02] for the proposed ACD 3D U-Net and [0.98, 1.01] for 3D nnU-Net.

**Fig. 7:**
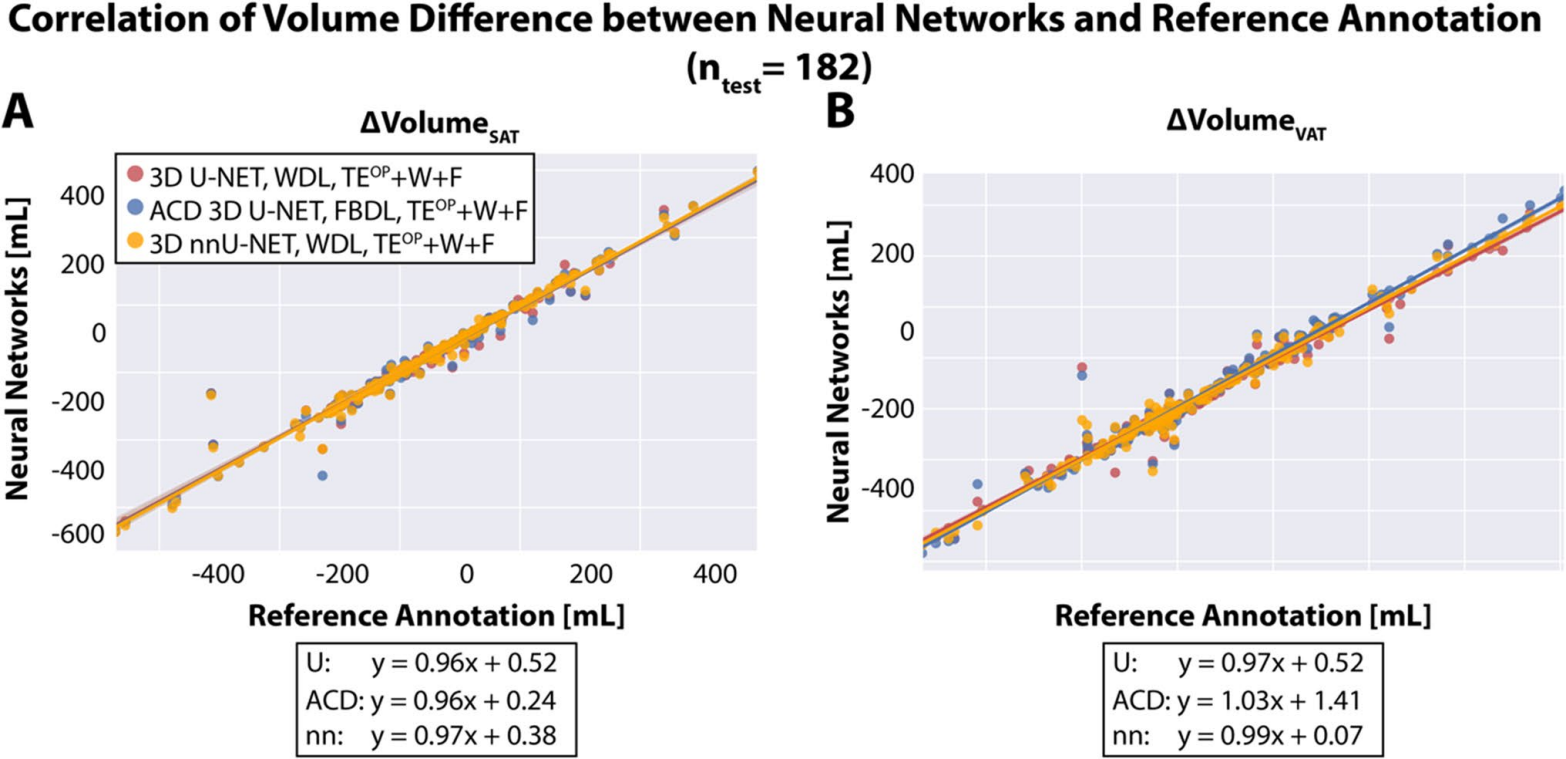
Correlation of abdominal SAT (**A**) and VAT (**B**) volume difference [mL] (between the first and second MRI scans) for results from 3D convolutional neural networks (3D U-Net [U], ACD 3D U-Net [ACD], and 3D nnU-Net [nn]) and reference annotations. Linear regression equations are shown

Figure 7 shows the correlation of SAT/VAT volume differences (between two MRI scans) obtained from the results by three 3D CNNs (3D U-Net, ACD 3D U-Net, and 3D nnU-Net) versus reference annotations. All three 3D CNNs achieved linear regression slopes close to 1, indicating high correlation of $\Delta$ Volume_SAT_ and $\Delta$ Volume_VAT_ between 3D CNN results and reference annotations.

## Discussion

In this study, we investigated automated abdominal SAT/VAT segmentation and volume quantification using 3D U-Net, ACD 3D U-Net, and 3D nnU-Net in a longitudinal MRI study using full-FOV volumetric multi-contrast inputs of TE^OP^, W, and F images and different loss functions (WDL or FBDL). Our proposed ACD 3D U-Net achieved excellent segmentation performance for the testing datasets (median DICE-SAT: 0.994/0.994, median DICE-VAT: 0.976/0.977, median FN: 0.6%/0.7%, median FP: 1.1%/1.0% in the first/second scan) and volume quantification (ICC-SAT: 0.998/0.997, ICC-VAT: 0.997/0.997, linear regression slope for SAT: 0.97/0.98, and linear regression slope for VAT: 1.03/1.02 in the first/second scan) with respect to the reference segmentations. Another key contribution of our work was to train and test 3D nnU-Net for segmenting abdominal SAT and VAT in a large cohort of adults with overweight/obesity at two time points. The trained 3D nnU-Net also showed high performance levels in terms of DICE-SAT (median: 0.992/0.993 for the first/second scan), DICE-VAT (median: 0.979/0.980), FN (1.1%/1.1%), and FP (1.2%/1.2%). When compared to 3D U-Net, the proposed ACD 3D U-Net and 3D nnU-Net achieved significantly better segmentation performance with higher median DICE-SAT and DICE-VAT scores, lower FN, and comparable FP. While ACD 3D U-Net and 3D nnU-Net achieved overall similar performance, 3D nnU-Net could be preferred due to its increased minimum performance levels, especially for DICE-VAT. All tested 3D CNNs achieved rapid inference times (< 4.8 s per 3D volume). 3D nn-UNet had the fastest inference time (< 2.5 s per 3D volume) among the tested CNNs. Note that these differences in inference times could be due to using different input data formats in the specific implementations, as 3D U-Net and ACD 3D U-Net used “h5” files, while nnUNet utilized “nii” files. In this study, 3D nnU-Net was the largest model with the largest number of trainable parameters, and therefore required a large dataset for sufficient training; this was satisfied by the datasets in our work. Considering the smaller number of trainable parameters and excellent performance in VAT segmentation, the proposed ACD 3D U-Net may have better generalization capability than 3D U-Net and 3D nnU-Net. Notably, our dataset included MRI scans from multiple scanners and multiple imaging sites, therefore providing further evidence regarding the generalization capability for the proposed ACD 3D U-Net and 3D nnU-Net.

Over a 6-month period, subjects in the HAT underwent two MRI scans to measure the change in abdominal VAT volume and distribution. By using the HAT data, one of our contributions in this work is to evaluate the 3D U-Net, the proposed ACD 3D U-Net, and 3D nnU-Net for analyzing longitudinal MRI data. There were changes in SAT and VAT volume between first and second MRI scans. The high correlation of $\Delta$ Volume_SAT_ and $\Delta$ Volume_VAT_ between reference annotations and results obtained using 3D CNNs shows that the changes in the adipose tissue volume between the first and second MRI can be accurately measured by the 3D CNNs investigated in this study. The first MRI scan was already able to capture the range of SAT/VAT characteristics for training the CNN models. Therefore, the CNN models could be trained using the first MRI scan and then successfully applied to the second MRI scan without additional training. High ICC and slopes close to unity to assess the agreement of volume quantification demonstrate the potential of the proposed ACD 3D U-Net and 3D nnU-Net to be applied consistently across multiple time points. This shows that the tools we developed here could potentially be generalized to large-scale and longitudinal studies for measuring and monitoring abdominal SAT/VAT volume.

The first and second MRI scans had identical MRI acquisition parameters. The list of these parameters includes but is not limited to: TE, TR, number of slices, slice thickness, in-plane resolution. The subjects were always positioned supine for both of the scans. Some scan conditions and characteristics such as SNR, breath-holding capability, liver position during breath-holding, and motion-induced artifacts may depend on the factors at the time of the scan, and therefore might differ between the first and second scans. Although these characteristics might vary, the tested 3D CNNs yielded excellent segmentation and volume quantification performance when trained using the first scan, and then tested on the second scan.

Our work included a systematic ablation study using the first MRI scan in 182 testing subjects. All tested CNNs yielded excellent SAT/VAT segmentation performance. Our results in the first ablation study regarding the ACD 3D U-Net showed that the ACD 3D U-Net structure improved the minimum, maximum, and median DICE-VAT, and yielded comparable DICE-SAT and median FP when compared to the 3D U-Net structure. In addition, for ACD 3D U-Net, FBDL improved the minimum DICE-SAT and DICE-VAT and reduced the maximum FN and FP when compared to WDL. FBDL and WDL led to similar performance levels for 3D nnU-Net. However, 3D nnU-Net with WDL required less time to train than with FBDL, therefore WDL could be preferred over FBDL for 3D nnU-Net. Note that WDL was optimized to be used together with 3D nnU-Net in its original published code implementation, whereas our code implementation for FBDL was not specifically tailored to be combined with 3D nnU-Net. We suspect this could play a role in increasing the training time for 3D nnU-Net with FBDL. Lastly, W and F images were the most crucial for feature extraction in abdominal AT as shown by the first ablation study for ACD 3D U-Net. Consistently, the worst segmentation performance in the ablation study was from the CNN using only TE^OP^ and TE^IN^ images. The addition of the TE^OP^ image to W and F images had incremental benefits. Our ablation study included input images with the most distinct fat/water contrast in a single image, as well as across different input images to improve the segmentation performance. Therefore, we did not consider TE^IN^ as an input image, as TE^IN^ has reduced fat/water contrast when compared to TE^OP^, W or F images.

Our training (n = 646), validation (n = 92), and testing (n = 182) datasets are among the largest reported in the literature. Using such large training/testing datasets strengthened the validity of the results and enabled statistical tests in this study. Compared to previous works for VAT segmentation in adults, our proposed ACD 3D U-Net and 3D nnU-Net achieved higher DICE-VAT values (median: 0.976 and 0.979) than the 2.5D FatSegNet with TE^OP^ + TE^IP^ inputs (DICE-VAT: 0.85) that focused on abdominal SAT/VAT quantification [16]. When compared to 3D Densely Connected Net with W + F patch inputs (Dice-VAT: 0.89) [24] that focused on whole-body SAT/VAT quantification, we reported higher DICE-VAT scores for the 3D CNNs in our study, including the 3D U-Net. The increase in DICE-VAT might be due to a few reasons: our work used a combination of multi-contrast inputs (TE^OP^ + W + F) and employed inputs with full-FOV volumetric coverage. This way, all our tested 3D CNNs were able to capture the global associations in SAT/VAT. Another previous work which employed full-resolution 3D nnU-Net (i.e., full-FOV coverage) cross-validated SAT/VAT segmentation of the body trunk (hip to cardiac apex) in 30 adults and achieved mean DICE-SAT/DICE-VAT of 0.981/0.947, respectively [32]. Different from this study, our work performed extensive training and testing in a larger cohort (n = 182) with longitudinal MRI, and reported better DICE-SAT and DICE-VAT. Note that the comparisons between whole-body versus abdominal SAT/VAT segmentation are not direct comparisons due to differences in slice coverage.

Our work was a retrospective study using HAT data to evaluate 3D CNNs to automatically segment and quantify SAT/VAT in the abdomen, rather than the whole body. There were several studies with slice coverage similar to our study [23, 46] that pointed out abdominal SAT/VAT, especially centered around L2-L3 or L4-L5 [47], might represent total SAT/VAT. As a result, the slice coverage in our study can be adequate for body composition analysis in the abdomen.

The data used in our work were originally acquired and processed for the HAT. Per HAT study aims, the 51 contiguous axial slices (255 mm in S/I direction) were selected from a single acquisition to cover as much of the abdomen as possible while maintaining consistent image and data quality. Analyzing a larger volume would require multiple acquisitions at different table positions in multiple breath-holds, which may result in the diaphragm and liver being in slightly different positions, complicating the process of matching the slice positions. Increasing the coverage would also increase the duration required to pre-process and perform reference annotation of SAT/VAT in the images, as there were manual steps (e.g., exclusion of the vertebrae) in the HAT study design; these aspects were not in the scope of our work. Note that this coverage of the abdomen is already markedly larger than many studies relying on cross-sectional imaging, with many previous studies using 1–20 slices [48–50]. The extension and evaluation of the 3D CNNs in this work to different volumetric coverage should be a topic for future investigation.

Our study had limitations. First, the investigated 3D CNNs require large memory due to full-FOV volumetric inputs. The high memory requirement could be a potential issue in automated whole-body SAT/VAT segmentation with full-FOV processing, in which the required GPU memory will be higher than abdominal SAT/VAT segmentation with image patch-based processing. Here, we used 3D nnU-Net with full-FOV inputs, but 3D nnU-Net can also be trained with smaller patches to alleviate the high memory requirements. Further research can explore the trade-offs between patch-based and full-FOV implementations of 3D CNNs. Second, this work used MRI scans acquired with a specific sequence and set of imaging parameters in a cohort with specific characteristics. The data characteristics are a current limitation due to the retrospective nature of using existing HAT data, but not a theoretical limitation. We plan to investigate the generalization of our 3D CNNs to different volumetric coverage and imaging characteristics. Further research is needed to analyze the generalizability of our proposed 3D CNNs to different study cohorts (e.g., children) and input images from other imaging sequences (e.g., T_2_-weighted images). Third, our longitudinal study data only had two time points separated by 6 months. Therefore, future work can further evaluate the proposed 3D CNNs in more time points over a longer time frame. Also, the differences in performance levels of previous works could be due to different subject characteristics, reference annotation guidelines, and evaluation methodology. The segmentation performance comparisons between our study and previous literature discussed here were based on reported numbers, and therefore cannot be considered a direct comparison with optimized code implementation and matched data characteristics.

In conclusion, ACD 3D U-Net and 3D nnU-Net achieved accurate segmentation and volume quantification of abdominal SAT/VAT using data from a longitudinal MRI study in a relatively large cohort, demonstrating the promising generalization capability and therefore the potential to use these 3D CNNs for analyzing and monitoring abdominal SAT/VAT longitudinally in adults with overweight/obesity.

### Supplementary Information

Below is the link to the electronic supplementary material.

Supplementary file1 (MP4 9719 KB)

Supplementary file2 (MP4 10,552 KB)

Supplementary file3 (DOCX 1009 KB)

## Data Availability

The data in this study was obtained from a clinical trial registered under the name “Habitual Diet and Avocado Trial (HAT)” with the unique identifier NCT03528031. The link is provided here: https://clinicaltrials.gov/ct2/show/NCT03528031. This study was a secondary analysis that used the data from the HAT. Data from the HAT is available by request from the source data publisher. Please refer to this paper for the primary analysis of the HAT data: Lichtenstein AH, Kris‐Etherton PM, Petersen KS, Matthan NR, Barnes S, Vitolins MZ, et al. Effect of Incorporating 1 Avocado Per Day Versus Habitual Diet on Visceral Adiposity: A Randomized Trial. Journal of the American Heart Association. 2022; 11(14):e025657. 10.1161/JAHA.122.025657.

## Abbreviations

CNN: Convolutional neural networks
3D: 3-Dimensional
SAT: Subcutaneous adipose tissue
VAT: Visceral adipose tissue
ACD: Attention-based competitive dense
WDL: Weighted Dice loss
FBDL: Frequency-balancing boundary-emphasizing Dice loss

## Material/code availability

The code for the 3D convolutional neural networks (CNNs) is available at https://github.com/HoldenWuLab/AT_Seg_3DCNN under an Academic Software License: ^©^ 2023 UCLA (“Institution”). The final weights for the trained 3D CNNs are available at https://github.com/HoldenWuLab/AT_Seg_3DCNN and can be used under a non-commercial, no derivatives Creative Commons (Attribution-NonCommercial-NoDerivatives 4.0 International) license.
