# Supplementary material for: Automated abdominal adipose tissue segmentation and volume quantification on longitudinal MRI using 3D convolutional neural networks with multi-contrast inputs
Source: MAGMA. 2024 Feb 1;37(3):491–506. doi: 10.1007/s10334-023-01146-3 (PMC11316694; doi:10.1007/s10334-023-01146-3)
Supplement: Supplementary file 3 — Supplementary file3 (DOCX 1009 KB) [file 10334_2023_1146_MOESM3_ESM.docx]

**SUPPLEMENTARY MATERIAL**

**Supplementary Video 1.** 3D renderings of the abdominal subcutaneous and visceral adipose tissue (SAT, VAT) segmentation mask outputs for the proposed ACD 3D U-Net with FBDL and TE^OP^+W+F for the two representative subjects shown in Figure 2. ACD: attention-based competitive dense, FBDL: frequency-balancing boundary-emphasizing Dice loss, TE^OP^: opposed-phase image, W: water image, F: fat image.

**Supplementary Video 2.** 3D renderings of the abdominal subcutaneous and visceral adipose tissue (SAT, VAT) segmentation mask outputs for the 3D nnU-Net with WDL and TE^OP^+W+F for the two representative subjects shown in Figure 2. WDL: weighted Dice loss, TE^OP^: opposed-phase image, W: water image, F: fat image.

*The videos are uploaded separately.*

**Supplementary Table 1.** Training time, inference time, and number of trainable parameters for all 3D convolutional neural networks in the ablation studies. ACD: Attention-based competitive dense, WDL: weighted Dice loss, FBDL: frequency-balancing boundary-emphasizing Dice loss, TE^OP^: opposed-phase image, TE^IN^: in-phase image, W: water image, F: fat image.

| **Network** | **Loss function** | **Inputs** | **Training time** | **Inference time** | **Trainable parameters** |
| --- | --- | --- | --- | --- | --- |
| 3D U-Net | WDL | TE^OP^+W+F | 22.6 hours | 65 ms/slice | ~22.4 M |
| ACD 3D U-Net | WDL | TE^OP^+W+F | 32.5 hours | 68 ms/slice | ~20.8 M |
| ACD 3D U-Net | FBDL | TE^OP^+ TE^IN^ | 32.6 hours | 75 ms/slice | ~20.8 M |
| ACD 3D U-Net | FBDL | W+F | 32.5 hours | 72 ms/slice | ~20.8 M |
| ACD 3D U-Net | FBDL | TE^OP^+W+F | 32.9 hours | 72 ms/slice | ~20.8 M |
| 3D nnU-Net | WDL | TE^OP^+W+F | 66.6 hours | 36 ms/slice | ~30.0M |
| 3D nnU-Net | FBDL | TE^OP^+W+F | 136.0 hours | 36 ms/slice | ~30.0M |


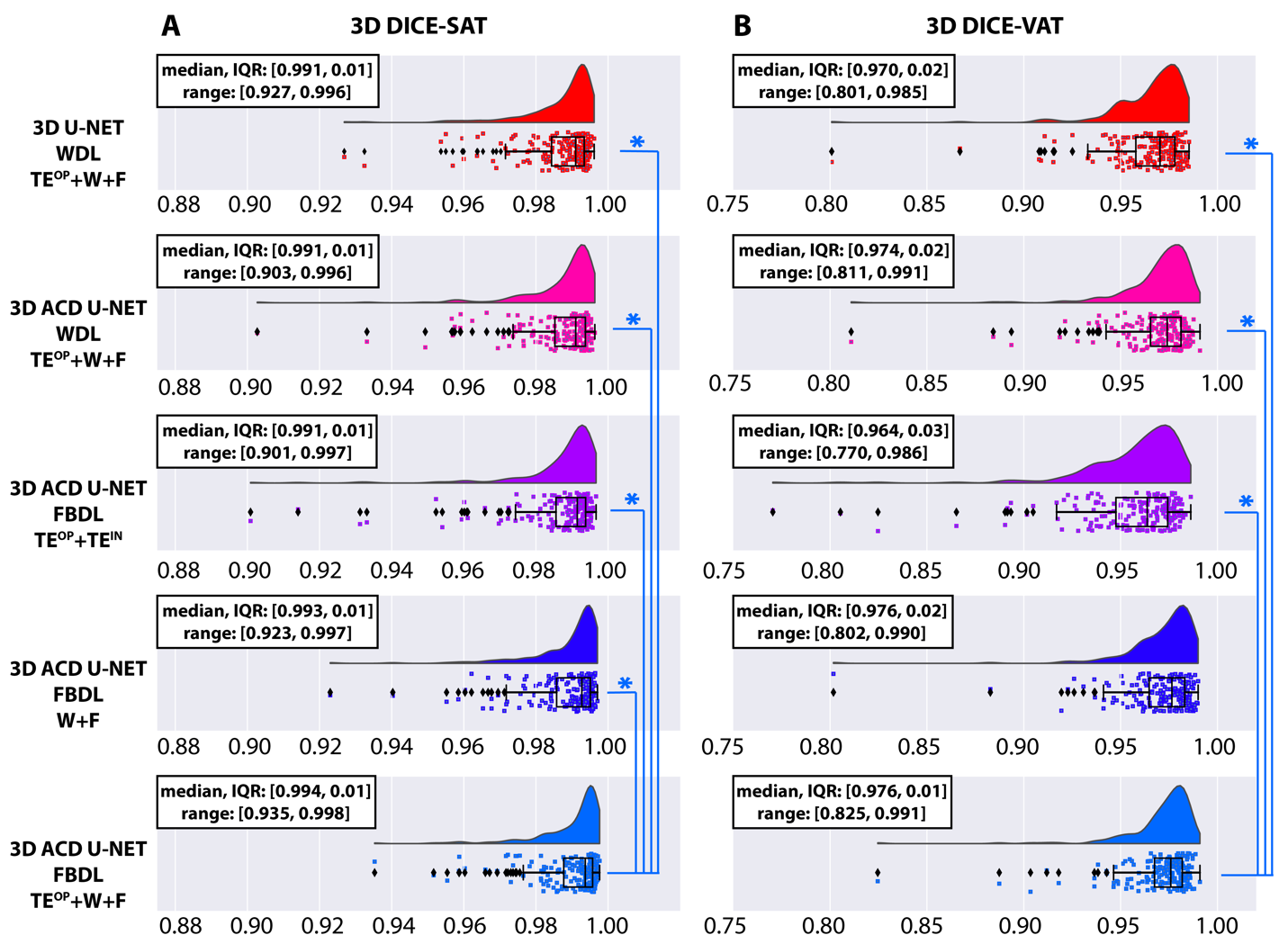


**Supplementary Figure 1.** Using the testing set (n=182) of the first MRI scans, **(A)** 3D DICE-SAT and **(B)** 3D DICE-VAT results for all five convolutional neural networks in the attention-based competitive dense (ACD) 3D U-Net ablation study are shown here in cloud and box/whisker plots. The median and interquartile range (IQR) values as well as the range of the DICE-SAT and DICE-VAT values were reported. The proposed ACD 3D U-Net with frequency-balancing boundary-emphasizing Dice loss (FBDL) and TE^OP^+W+F as inputs achieved better segmentation performance with higher median and minimum DICE-SAT and DICE-VAT scores. The * indicates statistically significant higher median scores for the proposed ACD 3D U-Net using Benjamini-Hochberg’s procedure for multiple comparison.


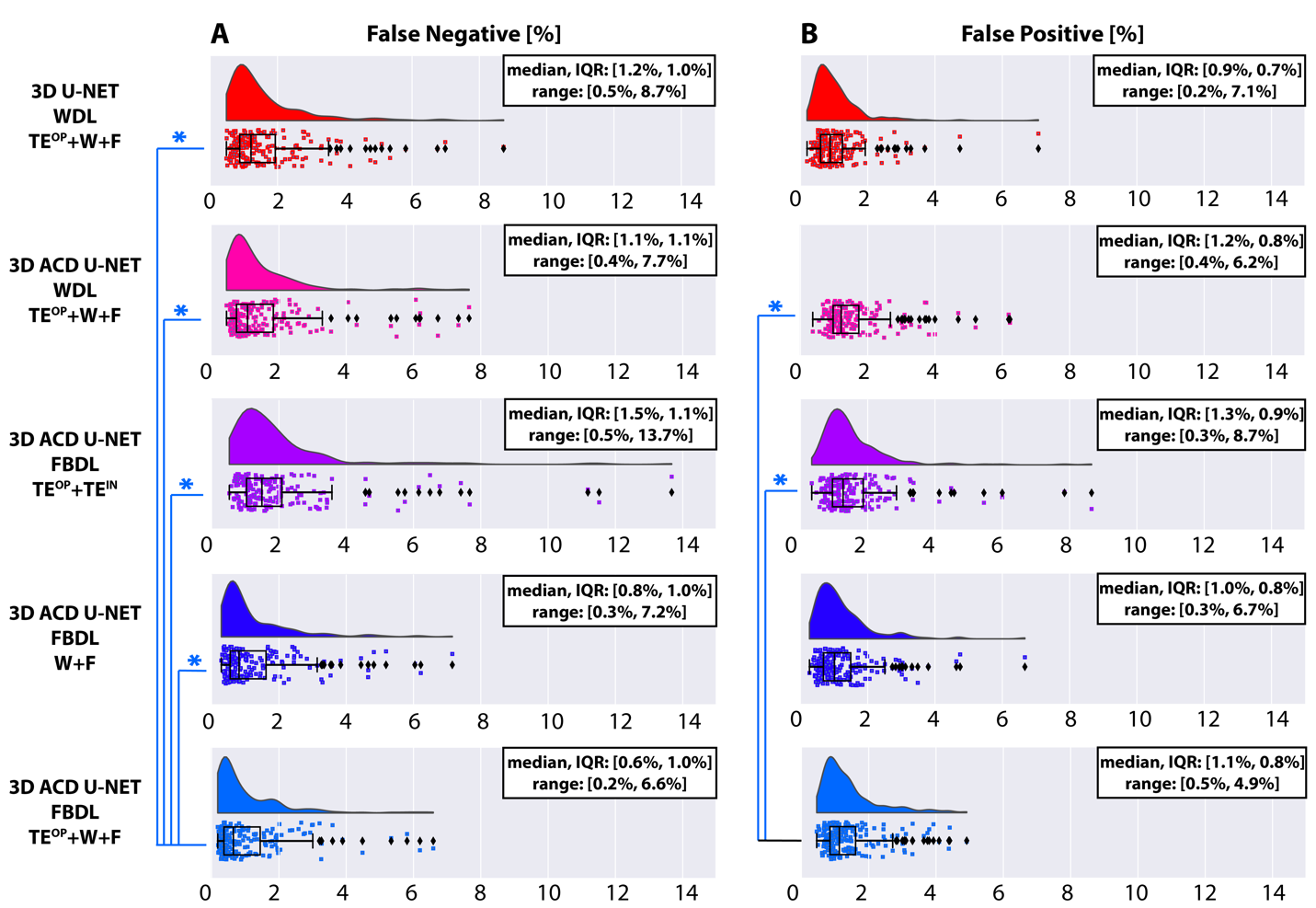


**Supplementary Figure 2. (A)** False negatives (FN) and **(B)** false positives (FP) for the testing set (n=182) of the first MRI scan using all five convolutional neural networks in the attention-based dense competitive (ACD) 3D U-Net ablation study are shown here in cloud and box/whisker plots. The median and interquartile range (IQR) values as well as the range of the FN and FP values were reported. The overall best performing neural network (lowest median FN and lowest maximum FN and FP) was the proposed ACD 3D U-Net with frequency-balancing boundary-emphasizing Dice loss (FBDL) and TE^OP^+W+F as inputs. The * indicates statistically significant differences in median scores for the proposed ACD 3D U-Net compared to other networks using Benjamini-Hochberg’s procedure for multiple comparison.


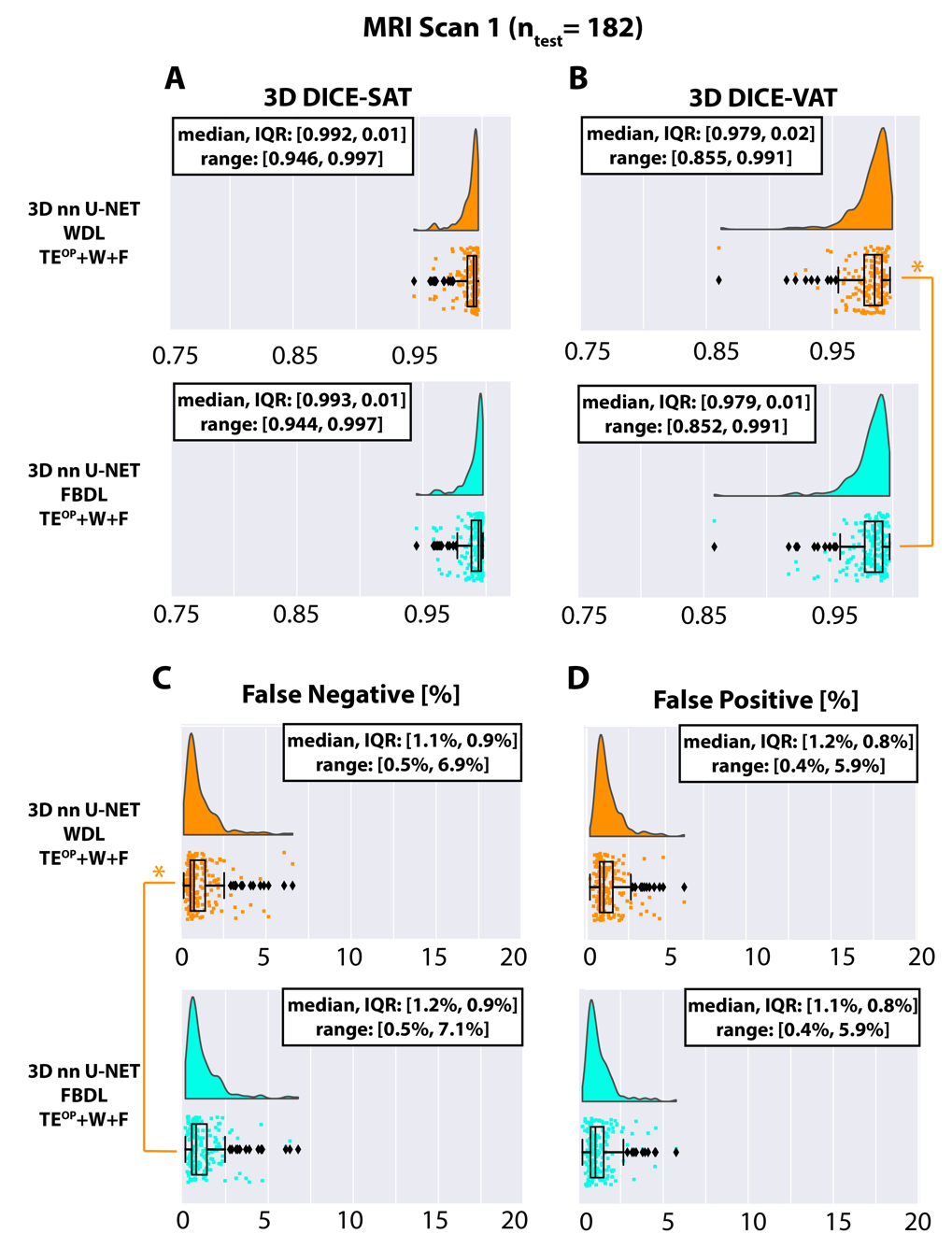


**Supplementary Figure 3.** Using the testing set (n=182) of the first MRI scans, results for 3D DICE-SAT **(A)**, 3D DICE-SAT **(B)**, false negatives **(C)**, and false positives **(D)** are shown for the ablation study of 3D nnU-Net trained with either WDL or FBDL using cloud and box/whisker plots. The * indicates statistically significant differences in median scores for 3D nnU-Net with FBDL versus WDL, using Benjamini-Hochberg’s procedure for multiple comparison. While 3D nnU-Net with WDL and FBDL did not have significant differences for DICE-SAT and FP, there were statistically significant differences in DICE-VAT scores and FN.
